# Proteomic-Based Approach Reveals the Involvement of Apolipoprotein A-I in Related Phenotypes of Autism Spectrum Disorder in the BTBR Mouse Model

**DOI:** 10.3390/ijms232315290

**Published:** 2022-12-04

**Authors:** Qi Li, Yaxin Shi, Xiang Li, Yuan Yang, Xirui Zhang, Lisha Xu, Zhe Ma, Jia Wang, Lili Fan, Lijie Wu

**Affiliations:** Department of Children’s and Adolescent Health, Public Health College, Harbin Medical University, Harbin 150081, China

**Keywords:** apolipoprotein A-I, autism spectrum disorder, proteomics-based approach, hippocampal tissue, phenotypes

## Abstract

Autism spectrum disorder (ASD) is a neurodevelopmental disorder. Abnormal lipid metabolism has been suggested to contribute to its pathogenesis. Further exploration of its underlying biochemical mechanisms is needed. In a search for reliable biomarkers for the pathophysiology of ASD, hippocampal tissues from the ASD model BTBR T+ Itpr3tf/J (BTBR) mice and C57BL/6J mice were analyzed, using four-dimensional (4D) label-free proteomic analysis and bioinformatics analysis. Differentially expressed proteins were significantly enriched in lipid metabolic pathways. Among them, apolipoprotein A-I (ApoA-I) is a hub protein and its expression was significantly higher in the BTBR mice. The investigation of protein levels (using Western blotting) also confirmed this observation. Furthermore, expressions of SphK2 and S1P in the ApoA-I pathway both increased. Using the SphK inhibitor (SKI-II), ASD core phenotype and phenotype-related protein levels of P-CREB, P-CaMKII, and GAD1 were improved, as shown via behavioral and molecular biology experiments. Moreover, by using SKI-II, we found proteins related to the development and function of neuron synapses, including ERK, caspase-3, Bax, Bcl-2, CDK5 and KCNQ2 in BTBR mice, whose levels were restored to protein levels comparable to those in the controls. Elucidating the possible mechanism of ApoA-I in ASD-associated phenotypes will provide new ideas for studies on the etiology of ASD.

## 1. Introduction

Autism spectrum disorder (ASD) is a group of neurodevelopmental diseases that manifests in early childhood and is characterized by social communication disorders, repetitive behaviors, and restricted interests. Patients may also experience cognitive impairment, sensory impairment, anxiety, and depression [1]. The prevalence of ASD has increased in recent years. Recently, the Centers for Disease Control and Prevention (CDC) statistics showed that ASD affects 1 in every 44 children [2]. Over the past decade, the total prevalence of ASD in Asian countries has increased from 0.15% to 0.3% [3]. Consequently, families and governments bear serious disease burdens and social problems, attracting widespread societal attention.

The etiology and pathogenesis of ASD remain unknown, and studies have focused on genomics, metabolomics [4], and imaging investigations [5]. Abnormalities in lipid metabolism contribute to the pathogenesis of ASD [6,7]. Lipid metabolism is essential for maintaining neuronal development and synaptic plasticity [8,9]. For brain function, lipids are essential. As reported in the literature, altered brain lipidome composition is found in the brains of patients with ASD, schizophrenia (SZ), and Down syndrome (DS) [10]. A recent study showed that a reduction in very low-density lipoprotein was correlated with apoprotein B (ApoB) in the serum of children with ASD [11]. The above-mentioned studies have suggested that lipid metabolism is associated with ASD; however, there are currently no reliable lipid metabolites that can be used as biomarkers for the mechanisms of the pathophysiology of ASD.

Due to the availability of limited tissue samples from patients with ASD, some studies have used mouse models that express traits relevant to autism to gain mechanistic insight. It has been shown that among these models, the BTBR T+ Itpr3tf/J (BTBR) mouse model shows most of the behaviors that are the core clinical manifestations in patients with ASD [12,13]. These mice also exhibit similar brain structures and functional abnormalities as patients with ASD, making them an ideal animal model for ASD. Accordingly, BTBR mice were selected for this study [14].

Proteomics is a valuable tool for identifying key molecules and pathways involved in various physiological and pathological processes [15]. It has been widely used to characterize molecular events in various disorders [16,17,18,19]. Furthermore, previous research has been constrained by the low resolution of the technology. In this study, we used a new-generation Tims ToF pro mass spectrometer with 4D label-free technology to perform mass spectrometry proteome analysis on the hippocampal tissue from BTBR mice. The hippocampus was chosen as it is the most directly associated with the core clinical phenotype of ASD. Significant enrichment results for all differentially expressed proteins showed that they were associated with lipid metabolism. Among them, apolipoprotein A-I (ApoA-I) was identified as a hub protein. However, there are no published studies on ApoA-I levels in patients with ASD. Herein, we first validated that ApoA-I in the hippocampal tissue of BTBR mice was differentially expressed, compared with that in control mice. Next, ApoA-I and its downstream molecules SphK2-S1P were found to be significantly upregulated using molecular biology methods. Surprisingly, ASD impaired phenotypes were improved via the use of a SphK inhibitor (SKI-II), which further confirmed that the ApoA-I pathway is highly correlated with ASD. ApoA-I downstream mechanisms were validated by observing changes in cognitive anxiety-related proteins, apoptosis, ion channels, and ASD-related signaling pathway proteins before and after the intervention. The potential mechanisms of action of ApoA-I in ASD have been elucidated to provide a theoretical basis for targeted interventions.

## 2. Results

### 2.1. Workflow for Discovering and Verifying the Roles and Mechanisms of ApoA-I-Related ASD 

**Figure 1:**
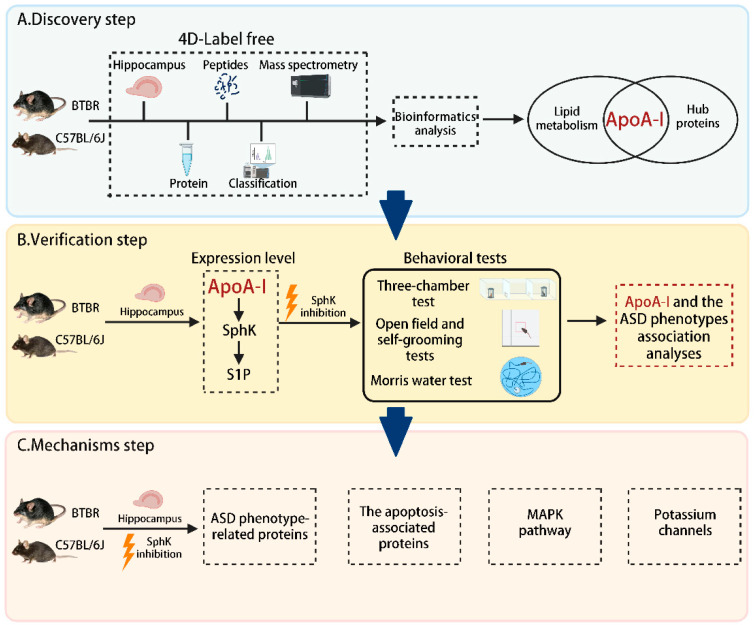
Overall workflow diagram for discovering and verifying the roles and mechanisms of ApoA-I-related ASD.

The experimental design of this study is illustrated in Figure 1. In the discovery step, proteins obtained from the hippocampus of male BTBR and C57BL/6J (C57) mice were analyzed using 4D protein quantification. By performing a bioinformatics analysis of differential proteins, ApoA-I was screened. For verification, a targeted ApoA-I study was set up to validate the proteomic results and confirm the expression levels of SphK and S1P in the ApoA-I pathway. By inhibition of SphK, the ASD-like behaviors in BTBR mice were improved, which suggests that the abnormal expression level of ApoA-I is highly correlated with ASD. Finally, the effects and possible mechanisms of ApoA-I-SphK in the pathogenesis of ASD were explored using molecular biological experiments.

### 2.2. Identification of Differential Proteins Using a 4D Label-Free Proteomic Analysis 

**Figure 2:**
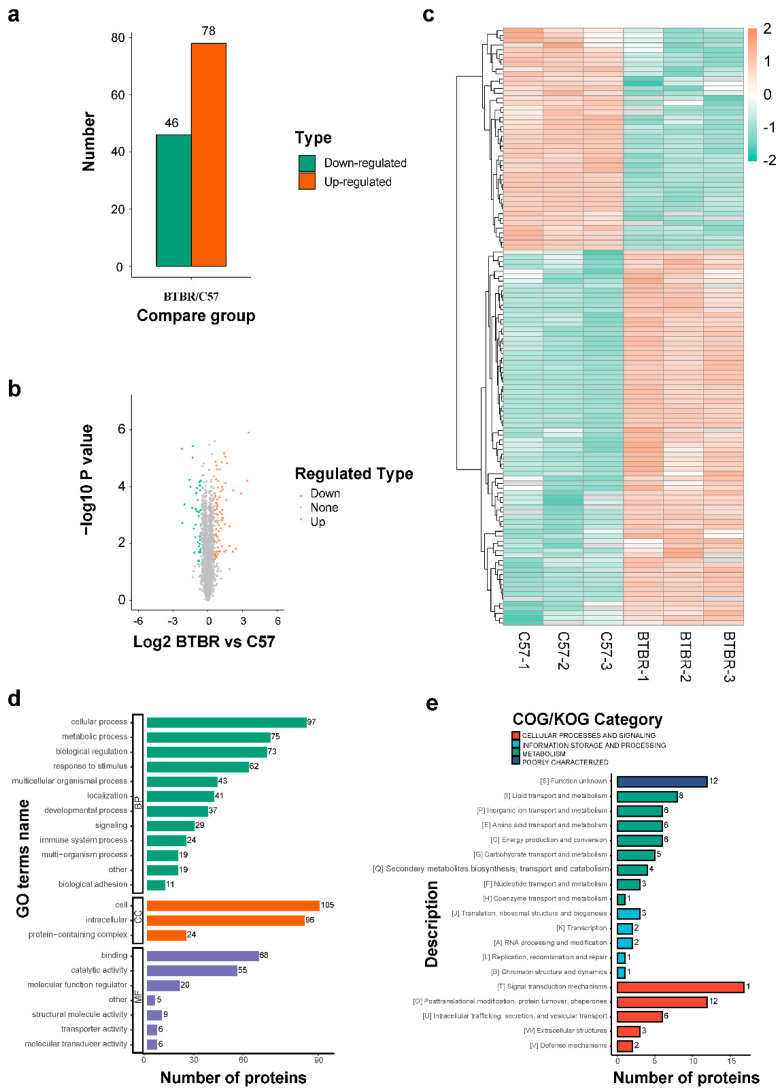
Identification of differential proteins (**a**–**c**) and GO/KOG functional classification (**d**,**e**). (**a**) The number of differentially expressed proteins was identified. Among them, 78 proteins were up-regulated and 46 proteins were down-regulated. (**b**) Volcanic map of differentially expressed proteins. Red represents upregulated proteins and green represents downregulated proteins. (**c**) Heatmap of differentially expressed proteins. Each small square represents a protein and the color indicates the level of expression. Red represents up-regulation and green represents down-regulation. The darker the color, the greater the expression. (**d**) GO functional classification of differentially expressed proteins. The abscissa represents the number of proteins and the ordinate represents the GO term name. (**e**) KOG functional classification of differentially expressed proteins. The abscissa indicates the number of proteins and the ordinate indicates the functional names in the different COG/KOG categories.

The hippocampal tissues from BTBR mice (n = 3, each sample was obtained from 10 mice) and C57 mice (n = 3, each sample was obtained from 10 mice) were subjected to 4D label-free proteomic analysis for differential protein screening. We focused on 124 proteins with significant changes in their expression levels (logFC < 0.5 and logFC > −0.5; *p* < 0.05), of which the expression levels of 78 proteins were increased and those of 46 proteins were reduced in the hippocampal tissue (Figure 2a). Differential proteins are presented in a volcano plot (Figure 2b), and a heatmap representation is shown in Figure 2c.

### 2.3. Bioinformatics Analysis for Differential Proteins

Functional classification was conducted by searching the Gene Ontology (GO) and Clusters of orthologous groups for eukaryotic complete genomes (KOG) databases to analyze the differentially expressed proteins involved in functional modulation in BTBR mice. According to GO functional classification, most proteins were enriched in cellular and metabolic processes (Figure 2d). Proteins enriched in lipid transport and metabolism signaling pathways were shown in the KOG function classification (Figure 2e). These results suggested that ASD was associated with aberrant lipid transport and metabolic proteins.

**Figure 3:**
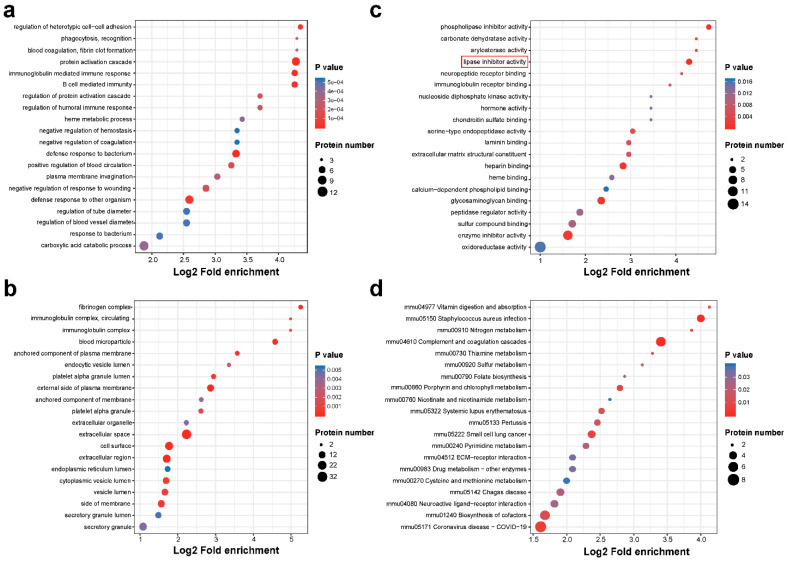
Functional enrichment analysis of differential proteins. The abscissa indicates log2 fold enrichment and the ordinate indicates different GO terms or KEGG pathways. The redder the circle, the smaller the *p*-value, and the larger the circle, the more proteins that are represented. (**a**) GO biological process enrichment analysis. (**b**) GO cellular component enrichment analysis. (**c**) GO molecular function enrichment analysis. (**d**) KEGG pathway enrichment analysis.

A GO/Kyoto Encyclopedia of Genes and Genomes (KEGG) functional enrichment analysis was used to analyze differentially expressed proteins (Figure 3). Figure 3a–c showed the top 20 enriched GO terms in the biological process, molecular function, and cellular component categories. The lipase inhibitor activity pathway (Figure 3c), which included proteins ApoA-I, Anxa4, Anxa5, and Anxa3, was significantly enriched according to the GO statistical distributions. ApoA-I, as the upstream regulator of S1P, has received significant attention based on our findings. S1P levels were elevated in the blood of children with ASD compared to the blood of controls in a previous metabolomic study [20]. In a KEGG functional enrichment analysis (Figure 3d), ApoA-I was strongly enriched in the PPAR pathway. Studies have shown that ASD patients with irritability, drowsiness/social withdrawal, and hyperactivity/non-compliance improved dramatically after receiving a PPAR agonist [21]. Thus, we selected ApoA-I as a research target to further explore its association with ASD.

**Figure 4:**
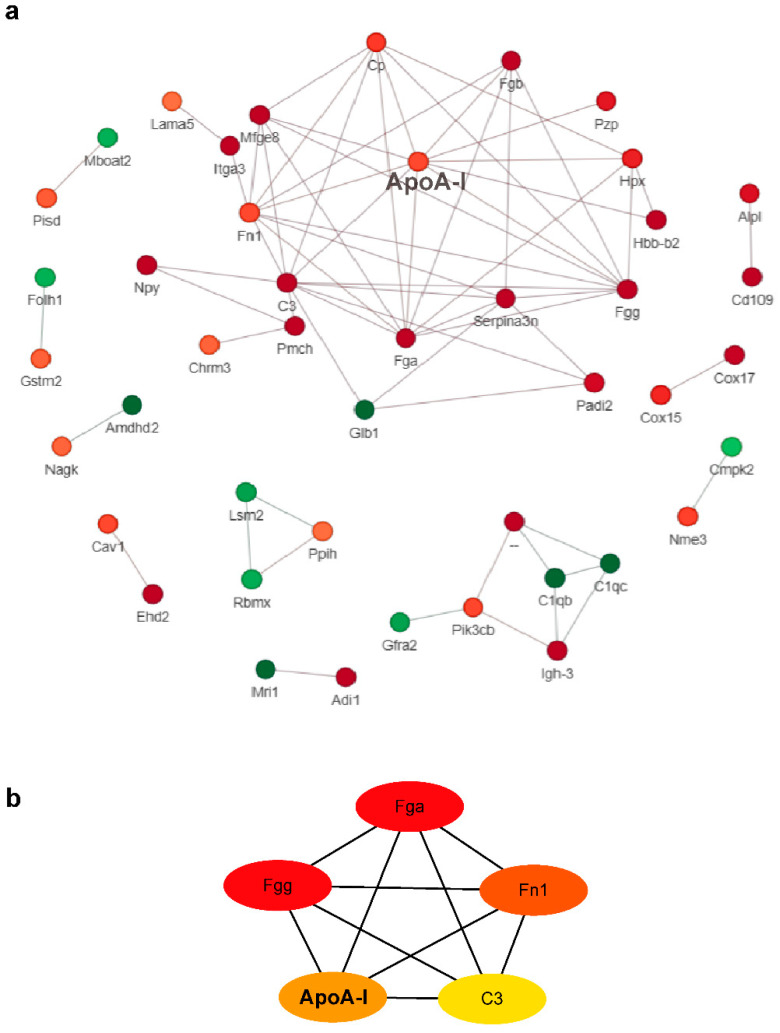
Protein–protein interaction network and screening of hub proteins. (**a**) Protein–protein interaction network of the top 50 differential proteins. (**b**) Protein–protein interaction network of the five screened hub proteins. The redder the color, the more protein connections there are in the network.

We used the STRING (version *V.11.0,* https://string-db.org/ accessed on 16 October 2022) protein interaction database to conduct an interaction analysis of all the differentially expressed proteins to identify key regulatory proteins. The top 50 high-confidence proteins in the interaction network (score > 0.7) were retained for visualization (Figure 4a). In the interactivity diagram, CytoHubba was used to further screen hub proteins, with the following being identified: FGA, FGG, FN1, ApoA-I, and C3 (Figure 4b). When examining the functional enrichment of all proteins, ApoA-I was not only the hub protein in the protein–protein interaction (PPI) diagram, but was also involved in the regulation of the lipid metabolism pathway. Therefore, the next step was to test our predictions experimentally, and the effect and mechanism of the ApoA-I protein on core phenotypes in ASD model mice were investigated. 

### 2.4. Expression of ApoA-I and Its Downstream Molecule SphK-S1P Expression in the Hippocampus of the ASD Mouse Model

**Figure 5:**
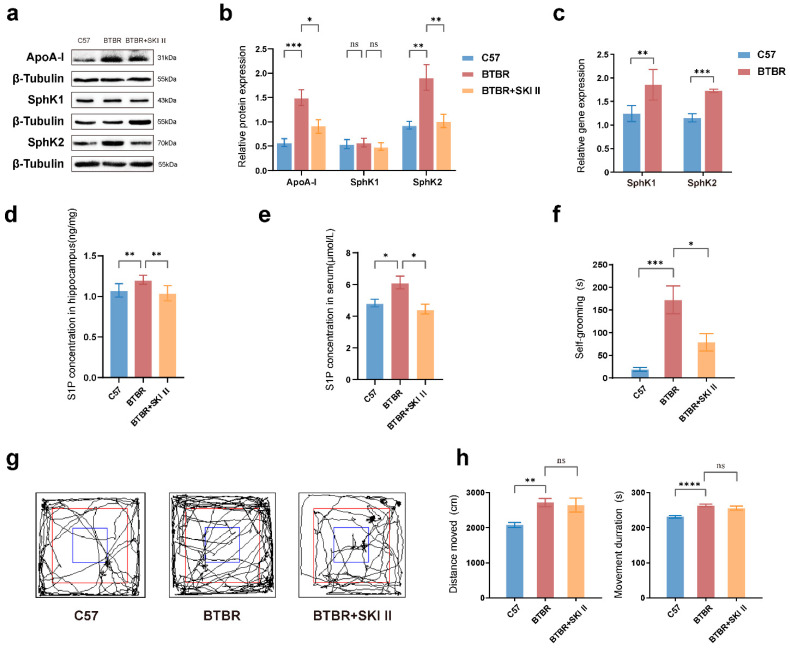
Examination of ApoA-I and its downstream molecule SphK-S1P expression and ASD behavioral phenotypes in BTBR mice before and after SKI II intervention. (**a**,**b**) Representative Western blotting bands and quantification of the ApoA-I/β-tubulin, SphK1/β-tubulin, SphK2/β-tubulin ratios in the ApoA-I pathway. (**c**) RT-qPCR quantification mRNA expression of SphK1 and SphK2. (**d**,**e**) S1P levels in the serum and hippocampus before and after SKI II intervention. (**f**) Time spent self-grooming before and after SKI II intervention. (**g**,**h**) Representative roadmap and quantification of distance moved and movement duration before and after SKI II intervention in the open field test. N = 8–12 per group. All data are shown in bar diagrams, which reflect the arithmetic mean ± standard error of the mean. * *p* < 0.05, ** *p* < 0.01, *** *p* < 0.001, **** *p* < 0.0001.

To further validate the bioinformatic analysis results, Western blotting was used to analyze the protein levels of ApoA-I. The results revealed that ApoA-I protein expression was significantly higher in the hippocampal tissues of BTBR mice than in the controls (Figure 5a,b; one-way ANOVA, *p* < 0.0008).

According to the literature, ApoA-I can increase S1P release by modulating SphK activity [22]. Subsequently, we found that the mRNA levels of SphK1/2 were higher in the hippocampal tissue of BTBR mice than in the controls (Figure 5c; SphK1, Welch’s *t* test, *p* = 0.0060; SphK2, Welch’s *t* test, *p* = 0.0004). SphK2 protein expression was higher in the hippocampal tissues of BTBR mice than in the controls (Figure 5a,b; *p* = 0.0036). The distribution of S1P in the hippocampus (Figure 5d; one-way ANOVA, *p* = 0.0088) and serum (Figure 5e; one-way ANOVA, *p* = 0.0299) was higher in the BTBR group than that in the control group. 

Protein detection revealed that SphK inhibitor (SKI-II) intervention in the BTBR group downregulated ApoA-I (Figure 5a,b; *p* = 0.0282) and SphK2 (Figure 5a,b; *p* = 0.0073), but not SphK1 (Figure 5a,b; *p* = 0.7750). S1P levels in the hippocampus (Figure 5d; *p* = 0.0019) and serum (Figure 5e; *p* = 0.0122) were dramatically reduced after the SKI-II intervention.

Therefore, BTBR mice showed an abnormal metabolism of apolipoproteins and their downstream metabolites, including high levels of ApoA-I and the corresponding downstream molecules, SphK2-S1P, in the hippocampus. Blockade of SphK downregulated the expression of ApoA-I and S1P.

### 2.5. Blockade of ApoA-I-Related Pathways Improved ASD Impaired Phenotypes 

**Figure 6:**
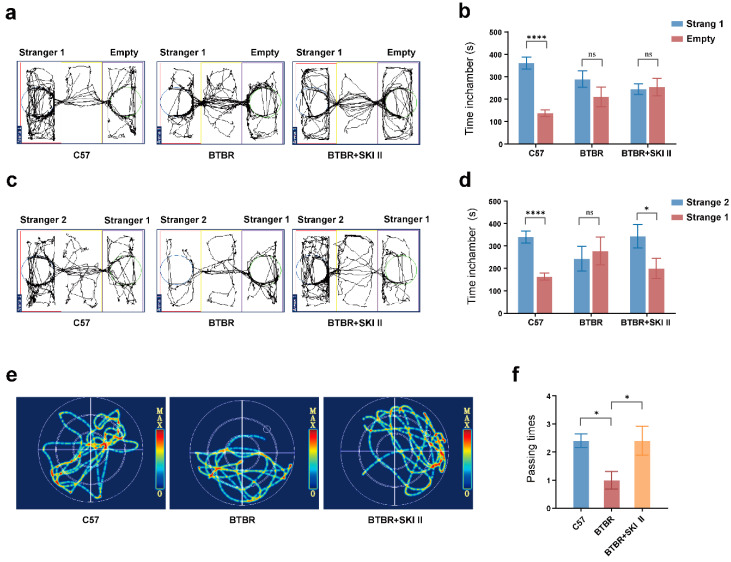
Blockade of ApoA-I-related pathways improved social ability, spatial learning and memory function in BTBR mice. (**a**,**b**) Representative roadmap and quantification of social preference in the three-chamber test. (**c**,**d**) Representative roadmap and quantification of social novelty in the three-chamber test. (**e**,**f**) Representative roadmap and number of target quadrant crossings (passing times) of the Morris water maze test. N = 12 per group. All data are shown in bar diagrams, which reflect the arithmetic mean ± standard error of the mean. * *p* < 0.05, **** *p* < 0.0001.

ASD-like behavioral phenotypes of BTBR mice have been reported previously [23]. In the three-chamber test, the control group mice showed a significant preference for unfamiliar mice compared with the object (Figure 6a,b; *t* test, *p* < 0.0001); however, BTBR mice showed social interaction deficits when choosing between the unfamiliar mice and the object (*p* = 0.2700). The social preference test, which examined whether the experimental mice preferred socially novel or familiar mice, revealed that the BTBR group showed reduced social novelty recognition (Figure 6c,d; *t* test, *p* = 0.6740), which differed from the control group (Figure 6c,d; *t* test, *p* < 0.0001). We further investigated the effect of SKI II on the autistic-like behavioral phenotypes of BTBR mice and found that it improved their impaired social preference (*t* test, *p* = 0.0178).

In the anxiety-related behavioral test, the open field and self-grooming tests, the BTBR group displayed greater distance moved (Figure 5g,h; one-way ANOVA, *p* = 0.0053), greater movement time (Figure 5h; one-way ANOVA, *p* < 0.0001), and more self-grooming (Figure 5f; Kruskal–Wallis test, *p* = 0.0001) than the control group. SKI II reduced self-grooming (Figure 5f; Kruskal–Wallis test, *p* = 0.0101) in BTBR mice. However, SKI II intervention did not affect the distance moved (Figure 5g,h; one-way ANOVA, *p* = 0.9276) or movement time (Figure 5g,h; one-way ANOVA, *p* = 0.5278) in BTBR mice.

The Morris water maze test was used to assess the effects of the SKI II on spatial learning and memory. The passing times of mice in the BTBR group were lower than those in the control group (Figure 6e,f; one-way ANOVA, *p* = 0.0441), whereas there was a significant increase in the passing times in the SKI II treatment groups, compared to that in the BTBR group (Figure 6e,f; one-way ANOVA, *p* = 0.0441). In summary, blocking the ApoA-I-SphK pathway in BTBR mice improved most of the ASD-related phenotypes in these mice.

### 2.6. Blockade of ApoA-I-Related Pathways Alters the Expression of Core Phenotype-Related Proteins

**Figure 7:**
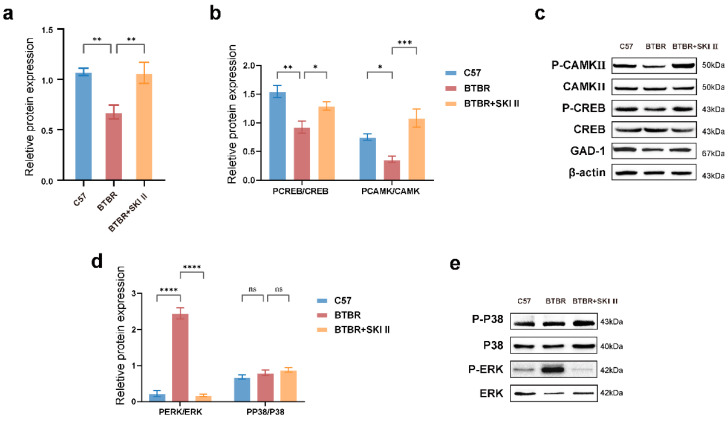
ApoA-I-related pathway regulates the expression of proteins related to anxiety, cognition and spatial learning function, as well as the MAPK pathway in the hippocampus of the mice. (**a**,**b**) Representative Western blotting bands and quantification of the anxiety-related protein GAD1/β-actin ratios. (**a**,**c**) Representative Western blotting bands and quantification of the cognition- and spatial learning-related proteins (P-CaMKII)/CaMKII, (P-CREB)/CREB ratios. (**d**,**e**) Representative Western blotting bands and quantification of the MAPK pathway protein P-P38/P38, P-ERK/ERK ratios. N = 8 per group. All data are shown in bar diagrams, which reflect the arithmetic mean ± standard error of the mean. * *p* < 0.05, ** *p* < 0.01, *** *p* < 0.001, **** *p* < 0.0001.

ApoA-I pathway-induced alterations in the hippocampus were reversed by the SphK inhibitor SKI II. The cognition- and spatial learning-related proteins CREB and CaMKII and the anxiety-related protein GAD1 were examined to explore the causes of impaired ASD-associated behavioral performance. Results were confirmed using western blot, which showed no alteration in the total protein levels of CREB and CaMKII in each group; however, the expression of phosphorylated CREB (P-CREB)/CREB (Figure 7a,c; one-way ANOVA, *p* = 0.0017), phosphorylated CaMKII (P-CaMKII)/CaMKII (Figure 7a,c; one-way ANOVA, *p* = 0.0444), and anxiety-related moleculeGAD1 (Figure 7a,b; one-way ANOVA, *p* = 0.0075) were lower in BTBR mice than in the control mice. In the BTBR + SKI II group (Figure 7a–c), the protein levels of P-CREB/CREB (*p* = 0.0441), P-CaMKII/CaMKII (*p* = 0.0008), and GAD1 (*p* = 0.0089) in the hippocampus reached levels comparable to those in the controls. Inhibition of the ApoA-I-SphK pathway by SKI II may improve behavioral performance regarding cognition-dominant learning, memory, and anxiety disabilities in ASD model mice.

### 2.7. Blockade of ApoA-I-Related Pathways Influenced MAPK Pathway-Associated Protein

The mitogen-activated protein kinase (MAPK) pathway is responsible for cognition, including learning and memory, and studies have shown that abnormal MAPK pathways are correlated with ASD [24]. In Figure 7d,e, the BTBR group showed significantly higher levels of phosphorylated ERK (P-ERK)/ERK (one-way ANOVA, *p* < 0.0001), a key MAPK signaling regulator, but there was no difference in the expression of phosphorylated P38(P-P38)/P38 (one-way ANOVA, *p* = 0.4898) compared with that in the control group. Subsequently, we examined MAPK pathway proteins after SKI II blockade in the BTBR + SKI II group (Figure 7d,e). SKI II downregulated P-ERK/ERK (one-way ANOVA, *p* < 0.0001), but did not regulate P-P38/P38 (one-way ANOVA, *p* = 0.6619). ApoA-I-induced alterations in the MAPK signaling pathways were reversed by the sphingosine kinase inhibitor SKI II.

### 2.8. Blockade of ApoA-I-Related Pathways Influenced Apoptosis-Associated Protein 

**Figure 8:**
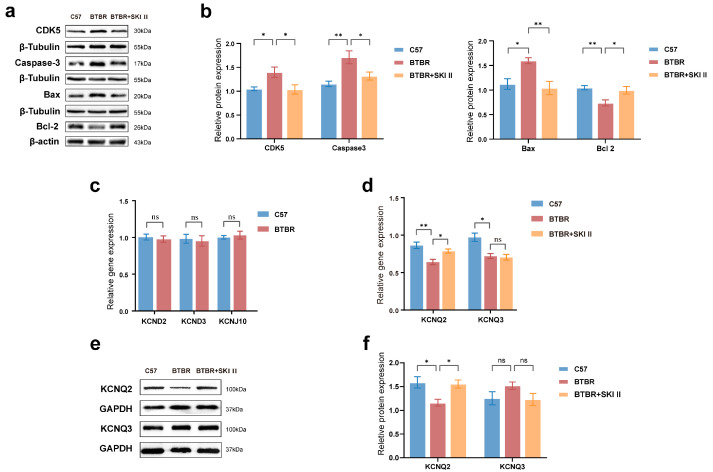
ApoA-I-related pathway regulates the expressions of proteins related to the apoptosis process and the KCNQ2 channel in the hippocampus of the mice. (**a**,**b**) Representative Western blotting bands and quantification of the apoptosis proteins, CDK5/β-tubulin, Caspase-3/β-tubulin, Bax/β-tubulin, and Bcl-2/β-actin ratios. (**c**) Quantification of mRNA expression of KCND2, KCND3, and KCNJ10 channels. (**d**) Quantification of KCNQ2, and KCNQ3 mRNA expression. (**e**,**f**) Representative Western blotting bands and quantification of the M-channel proteins, KCNQ2/GAPDH, and KCNQ3/GAPDH ratios. N = 8–10 per group. All data are shown in bar diagrams, which reflect the arithmetic mean ± standard error of the mean. * *p* < 0.05, ** *p* < 0.01.

To investigate whether high ApoA-I levels were associated with apoptosis, we measured the expression levels of apoptosis-associated proteins, including Caspase-3, Bax, Bcl-2, and cyclin-dependent kinase 5 (CDK5). Compared to the control group, Figure 8a,b showed that the expressions of CDK5 (one-way ANOVA, *p* = 0.0495), pro-apoptotic Caspase-3 (one-way ANOVA, *p* = 0.0077), and Bax (one-way ANOVA, *p* = 0.0164) increased, whereas those of the anti-apoptotic protein Bcl-2 (one-way ANOVA, *p* = 0.0055) decreased in the BTBR group. Compared with the BTBR group, in the BTBR + SKI II group (Figure 8a,b), CDK5 (one-way ANOVA, *p* = 0.0417), Caspase-3 (one-way ANOVA, *p* = 0.0471), and Bax (one-way ANOVA, *p* = 0.0092) were downregulated, whereas Bcl-2 (one-way ANOVA, *p* = 0.0215) was upregulated. All aberrantly regulated apoptosis-related proteins in the hippocampus of BTBR mice improved following SKI II intervention.

### 2.9. The Improvement of ASD Core Phenotypes by Blockade of the ApoA-I-Related Pathway May Be Associated with KCNQ2 Channels

Potassium channel dysfunction is relevant to the pathophysiology of ASD, particularly with regard to brain function abnormalities and neuronal excitability alterations [25]. The expression of KCND2, KCND3, KCNJ10, KCNQ2, and KCNQ3 mRNA in the hippocampus of BTBR and control mice was measured by RT-qPCR to confirm the level of ASD-related potassium channels. Figure 8c shows that the mRNA expression levels of KCND2 (*t* test, *p* = 0.6518), KCND3 (*t* test, *p* = 0.7403), and KCNJ10 (*t* test, *p* = 0.6101) were not significantly different between the BTBR and control groups; only KCNQ2 (Figure 8d; one-way ANOVA, *p* = 0.0015) and KCNQ3 (Figure 8d; Kruskal–Wallis test, *p* = 0.0143) expression levels were downregulated in BTBR mice. The potassium channel subunit KCNQ2 predominantly assembles with homologous KCNQ3 to form the “M” channel, which is mainly expressed in the hippocampus and cortex. Surprisingly, after SKI II intervention between the groups, both the protein (Figure 8e,f; one-way ANOVA, *p* = 0.0387) and mRNA (Figure 8d; one-way ANOVA, *p* = 0.0309) levels of KCNQ2 were upregulated in the BTBR+SKI II group compared to the BTBR group, and yet there were no significant differences in KCNQ3 mRNA (Figure 8d; one-way ANOVA, *p* > 0.9999), or protein levels (Figure 8e,f; one-way ANOVA, *p* = 0.2279). This demonstrates that the abnormally functioning M channels were mainly mediated by downregulated KCNQ2, which could be rescued by altering ApoA-I. However, the heteropolymerization of KCNQ3 showed no change after the intervention. Abnormal KCNQ2 channel function is strongly associated with ApoA-I-related pathways.

## 3. Discussion

ASD is a neurodevelopmental disorder of unknown etiology. In this study, we focused on the quantitative proteomic analysis of hippocampal tissue from an ASD model, BTBR mice. We used bioinformatic approaches and screened for ApoA-I, a protein that may have a close relationship with ASD. A targeted ApoA-I study was set up, using molecular biology experiments to validate the above-mentioned observations. The abnormal expression levels of ApoA-I signaling downstream molecules SphK and S1P were also confirmed in this study. The SphK inhibitor (SKI II) was subsequently used as an intervention, and behaviors were observed to verify the effect of ApoA-I-related pathway blockade in ASD and the improvement of ASD-associated core phenotypes. Moreover, the mechanisms of ApoA-I action on ASD were elucidated, which were found to involve cognitive proteins, apoptotic proteins, MAPK signaling pathways, and potassium channels. 

Neurodevelopmental and neurodegenerative disorders are characterized by abnormal lipid metabolism and accumulation [26]. Patients with ASD exhibit abnormal lipid metabolism [27]. We arrived at the same conclusion using the BTBR mice. A bioinformatic analysis revealed that differentially expressed proteins were significantly enriched in pathways associated with lipid metabolism. Among the differentially expressed proteins, ApoA-I is a hub protein, but its effects on ASD have not been reported. Western blotting analysis revealed high levels of ApoA-I in the BTBR mouse model of ASD. To our knowledge, this study is the first to investigate the effects of ApoA-I in an ASD model. It has been reported that ApoA-I expression in AD mice is considerably altered [28]. The ApoA-I level was elevated in an epileptic rat model after traumatic brain damage and its level was considerably lowered after spinal cord injury. In response to neuronal injury, ApoA-I is increasingly expressed and secreted, which is a self-protective mechanism [29]. Consistent with our finding that ApoA-I was upregulated in the hippocampus of the BTBR mice, we speculated that the alteration in ApoA-I may be a self-protection strategy for the injured system within the brain of those with ASD.

ApoA-I, a high-density lipoprotein (HDL) apolipoprotein, is a critical factor in the formation of HDL in the blood [22]. ApoA-I expedites the production and release of S1P via SphK [30]. Our study is the first to report that SphK2 downregulates ApoA-I and S1P levels in the hippocampus and serum of BTBR mice. This is a further exploratory step based on our previous finding that S1P levels were upregulated in the serum of children with ASD [20].

S1P is produced by SphK type 1 and 2. After the SphK intervention, we examined the behavioral alterations in BTBR mice to explore the ApoA-I downstream pathways, S1P levels, and expression of cognitive- and social ability-linked proteins. In the behavioral analysis, the BTBR group demonstrated a significant loss in social ability, repetitive and stereotyped behaviors, cognitive dysfunction, and anxiety behaviors compared with the control group, which is highly consistent with previous investigations [31]. It is gratifying to observe that ApoA-I and S1P levels decreased after the intervention, and ASD-like behavioral phenotypes were significantly recovered. Our experimental results were consistent with those in the ASD model with VPA rats [32]. It suggests that ApoA-I and downstream SphK regulate ASD behavioral phenotypes, including cognition, spatial learning and memory, and anxiety.

In the brain, GAD1 is present in GABAergic neurons. ASD is also accompanied by dysregulation of the GABA energy system. The GABA metabolic pathway is one of the causes of anxiety in VPA-induced ASD models [33,34]. In addition to this, reduced glutamate release is detected in adult BTBR mice [35]. Our findings were consistent with previous perspectives; in the hippocampus of BTBR mice, the GAD1 protein was downregulated. A decrease in GAD1 levels may lead to a decrease in inhibitory GABA signaling, resulting in hippocampal excitatory/inhibitory imbalance, abnormal synaptic plasticity, and abnormal formation of the neural network, thereby interfering with hippocampal function [33]. Furthermore, the excitation of GABA receptors reversed repetitive and stereotyped behaviors in two ASD models [36], and the differences in GABAergic function underlie the sensory neurobiology of autism [37]. Accordingly, the increased ApoA-I-SphK levels in ASD model mice may be involved in the occurrence and development of the downregulation of GAD1 in the hippocampus, potentially reducing the production of GABA in the brain. Taking the reduced GAD1 level as our starting point, we could further investigate the correlation between the aberrant engagement of ApoA-I-SphK metabolic and GABA energy systems.

The hippocampus is a critical integration center for learning and memory [38]. Recent research has revealed that there are abnormalities in the hippocampus in ASD patients from childhood to adolescence [39]. The Ca^2+^/calmodulin-dependent kinase (CaMKII) pathway and its downstream molecule, the cAMP response element binding (CREB) protein, play important roles in learning and memory in the hippocampus [40]. In a neurodegenerative disorder model, calmodulin (CaM) levels were found to be increased, whereas phosphorylated CREB levels were significantly decreased; although, CaMKII and CREB total protein levels did not change [41]. In both the prefrontal cortex and peripheral blood mononuclear cells, pCREB levels were found to be reduced in patients with Alzheimer’s disease [42]. The findings of the cognitive behavioral experiments, Morris water maze, and three-chamber task with BTBR mice were consistent with the decreased protein levels of phosphorylated CREB and phosphorylated CAMKII in the hippocampus, indicating that learning, memory, and social disorders in BTBR mice may be caused by the downregulation of phosphorylated CREB and phosphorylated CAMKII.

The MAPK/ERK signaling pathway is highly relevant to neurodevelopmental disorders, especially ASD [24]. The blockade of this pathway can significantly affect cognitive domains, including learning and memory. The three main conventional MAPK pathways are the ERK, p38, and JNK pathways. We provide evidence that in the hippocampus of ASD BTBR mice, the high levels of both ApoA-I and downstream SphK upregulate the phosphorylation of ERK1/2, but not P38 in the MAPK signaling pathway, a result that is consistent with previous findings [43]; although, the activation of the signaling network of p38MAPK has also been confirmed in ASD [44]. However, the results we obtained are consistent with the literature, which indicates that hyperactivation of the ERK pathway has been validated in BTBR mice [45]. Moreover, the dysregulation of pivotal ERK/MAPK signaling may contribute to the pathogenesis of ASD [46]. Therefore, our findings indicated that ApoA-I-SphK-ERK may significantly influence ASD-like behaviors and it may be a new therapeutic target for ASD.

In the hippocampal tissues of BTBR mice, we found alterations in the pro-apoptotic protein caspase-3, which has been identified as a key mediator of physiological neuronal apoptosis [47]. The cell lymphoma-2 (Bcl-2) family proteins participate in cell apoptosis and play an important role in regulating neuronal apoptosis and axonal degeneration [48]. The binding of cyclin I to CDK5 has been proposed to exertan anti-apoptotic function, which is possibly mediated by the activated MEK/ERK pathway and upregulated Bcl-2 and Bcl-xL expressions [49]. The reactivation of the cell cycle is considered an important feature of neurological disorders [50]. CDK5 is abundantly expressed in the nervous system and regulates learning and memory, neuronal development, synaptic transmission, and homeostatic plasticity [49]. Our results have shown significant abnormalities of P-ERK and CDK5 in the brains of BTBR mice, which could be rescued by SKI-II intervention. In contrast to our study, other studies have demonstrated that ERK1/2 and CDK5 are both activated in the hippocampus of VPA rats [51], possibly because different ASD models have different variations. Targeting the ApoA-I-SphK pathway will shed new light on therapeutic interventions. 

Potassium channels play a key role in the modulation of neuronal excitability and synaptic activity. Studies have shown that genetic mutations in ion channels are involved in ASD pathogenesis [52]. We examined mRNA alterations in ASD pathogenesis-related potassium channels in the hippocampus of BTBR mice, including voltage-gated potassium channels (KCNQ2 and KCNQ3) [53,54], A-type potassium channels (KCND2) [55], and inwardly rectifying potassium channels (KCNJ10) [56]. We observed that only KCNQ2 was upregulated in the BTBR group and that it was subsequently regulated by SphK. The KCNQ2/3 heteromeric channel has unique kinetic properties associated with the native M-current, which is strongly associated with epilepsy and ASD comorbidities [57], as well as a spectrum of developmentally regulated diseases in the brain [58]. It has been identified that KCNQ2/3 loss-of-function (LoF) or gain-of-function (GoF) negatively affects neuronal activity [59] in ASD patients [60,61], cell lines, and animal models [62]. We demonstrated that the SphK inhibitor could rescue the KCNQ2 protein expression in BTBR mice, indicating that ApoA-I-SphK has a potential positive regulatory role in the regulation of KCNQ2/3 and ASD-like phenotypes. In this study, we elucidated the effects and possible mechanisms of ApoA-I in the ASD phenotypes and provided new ideas for the clinical intervention of ASD. However, the current limitation in the literature is that in the lipid metabolic pathway, only ApoA-I has been studied intensively. It is necessary to further validate and investigate the post-translational modifications based on differentially expressed proteins in our proteomic analyses. Additional ASD mouse models can be used for future experimental validation. We hope that more studies related to lipid metabolism are undertaken, resulting in new avenues for intervention and therapy in patients with ASD.

## 4. Materials and Methods

### 4.1. Experimental Animals

BTBR T+ Itpr3tf/J (BTBR) mice were bred at the Jackson Laboratory as a model for ASD. C57BL/6J mice were bred at the Beijing Vital River Laboratory Animal Technology Co., Ltd. To exclude possible changes in social behavior due to unequal confounding of each group, one mouse from each group was placed in a cage in a controlled environment (12 h light/dark cycle, 21 ± 1 °C; 55 ± 5% humidity) with food and water available ad libitum. All experimenters were blinded to treatment conditions and experimental groupings.

### 4.2. Protein Extraction and Data Quality Control

Equal amounts of protein from each sample were digested, and the lysis solution was adjusted to the same protein concentration. Samples were lysed by ultrasonography and centrifuged to remove cell debris (4 °C, 10 min at 12,000× *g*). Protein concentration was determined using a BCA kit (Thermo Scientific, Waltham, MA, USA). Regarding peptide length distribution, most of the peptides ranged between 7 and 20 amino acids. Regarding peptide number distribution, in most proteins, more than two peptides were involved. When the proteins were quantified, one peptide per protein-specific peptide (or spectrum) enhanced the accuracy and credibility of the quantification results. Regarding protein coverage distribution, the protein coverage was less than 20% for most of the proteins.

### 4.3. Bioinformatics Analysis

#### 4.3.1. Differential Protein Analysis

The differential expression of proteins was identified by 4D label-free proteomic analysis. The screening conditions were set as follows: fold change of 1.5 and *p*-value of 0.05. The volcano map and heatmap were drawn by R package ggplot2 [63] and R package pheatmap [64], respectively.

#### 4.3.2. GO/KOG Function Classification

Gene Ontology (GO) is a tool used for bioinformatic data analysis that can be used to comprehensively describe the properties of genes and gene products in organisms. GO has a total of three ontologies, including molecular function, cellular component, and biological process, which explain the biological roles of proteins from different perspectives. The differentially expressed proteins were analyzed using eggNog-Mapper software (version 2.0) based on the EggNoG database. The GO ID was obtained from the annotation result of each protein; after this, the protein was classified based on the cellular component, molecular function, and biological function. A statistical analysis was performed on the distribution of differentially expressed proteins in GO secondary annotations.

COG, Clusters of Orthologous Groups of proteins (http://www.ncbi.nlm.nih.gov/COG/) accessed on 7 May 2021, stands for “homologous protein cluster”. COG was divided into two groups, one with prokaryotes and the other with eukaryotes. Clusters of orthologous groups (COG) and KOG were used to define orthologs in prokaryotes and eukaryotes, respectively. By database comparison and analysis, COG/KOG functional classification statistics were performed for differentially expressed proteins.

#### 4.3.3. Functional Enrichment Analysis

To obtain the enrichment of differentially expressed proteins for all the identified proteins, we identified enriched Encyclopedia of Genes and Genomes (KEGG) pathways by applying a two-tailed Fisher’s exact test; it was considered significant when the *p*-value was <0.05.

#### 4.3.4. Protein–Protein Interaction (PPI) Network Analysis

The STRING database was used for analyzing the interaction between differentially expressed proteins. A confidence score of >0.7 (high confidence) was considered as a cut-off value, and the top 50 proteins in the interaction relationship were visualized by using the R package networkD3 to obtain the PPI network. Subsequently, the CytoHubba plugin of the Cytoscape software (version 3.9.0) was used to further screen Hub proteins.

### 4.4. Animal Intervention

SKI II is a sphingosine kinase inhibitor that reduces S1P levels in the serum and brain when given intraperitoneally [65]. Four-week-old male BTBR mice were randomly divided into the SKI II intervention group (BTBR + SKI II group) and the model group (BTBR group), with 8–12 mice in each group. Four-week-old wild-type male C57BL/6J mice were taken as the normal control group (C57 group), with 8–12 mice in each group. The BTBR + SKI II group’s mice were injected once in the intraperitoneal cavity at a dose of 50 mg/kg once every 2 days for a total of 7 times with the SKI II (Selleck, S7176) mixture fluid, while the BTBR and C57 groups were injected with the vehicle in parallel. 

### 4.5. Analysis of Behavior

The experimental environment was kept quiet throughout testing. Before testing, the animals were exposed to the environment for at least 2 days. All of the behaviors were evaluated blindly. Two weeks after SKI II (or vehicle) intervention, the open field behavior test, the three-chamber test, the hair grooming test, and the Morris Water Maze test were performed on the mice. At the end of each test session, 75% alcohol was used to clean the inner wall and bottom of the arena to eliminate the residual urine and odor of the tested mouse. All behaviors were evaluated blindly.

#### 4.5.1. Open Field Test

The open field analysis was used to measure anxiety-like behavior in mice. The test was performed in a quiet environment using the SMART 3.0 opening experiment system. The experimental field was a 45 cm × 45 cm × 40 cm open box made of black polycarbonate, and a far-infrared camera was placed above the box. Mice were placed in the center of the box for simultaneous imaging and timing. Each mouse was allowed to explore the chamber for 10 min and the trajectory, time, and distance of the mice were recorded.

#### 4.5.2. Three-Chamber Test 

The three-chamber test was used to measure the sociability of the mice. During the habituation phase, mice were placed in the middle of the three-chamber apparatus for 10 min. The three-chamber apparatus is a box with three chambers, each area of the chamber is 20 cm × 40 cm, and the chambers on the left and right sides each contain empty barred cylinders on both sides. During sociability testing, the cylinders were filled with visual and olfactory cues so that the stimulus mice can observe and smell each other. The test mice and stimulus mice were able to directly communicate with each other through the cylinders. When the test animals were within 2 cm of a stimulus mouse in the cylinder, this was considered as evidence of social behavior. The social ability test began after the adaptation phase and the stranger mouse 1 (which the test mice did not recognize) was placed in the cylindrical mouse cage in the left chamber. The empty cage was placed in the right chamber, and the test mice were placed in the central chamber. In addition, the movement and activity time in each box were recorded by the camera for 10 min. This phase tested the preference of the test mice for engaging with the stranger mouse in comparison with the empty cage. Then, the social preference test began and the cage with mouse 1 was switched to the right chamber, while the cage with mouse 2 was placed in the left chamber. The movement and activity time of the test mouse were recorded by a camera for 10 min. This phase tested the preference of the test mice for engaging with the new, stranger mouse in comparison with the familiar mouse.

#### 4.5.3. Hair Grooming Test

Experiments were performed to test the level of stereotyped behavior in mice. Throughout the grooming chain, various forms of strokes and licks were all marked as grooming. Mice were placed into an empty standard cage for 10 min, and subsequently recorded. The duration spent grooming over 10 min was calculated during which the environment remained quiet.

#### 4.5.4. Morris Water Maze Test 

The Morris water maze test was used to assess cognitive function. Mice were tested at P42–P46 and the experimental equipment used included the WMT-100 Morris water maze automatic analysis system, with a 160 cm diameter tank carrying a far-infrared camera. The sink was divided into four quadrants, and an 8cm × 8cm platform was placed on the third quadrant. The sink was filled with an opaque liquid at a temperature of 22 ± 1 °C. The liquid was made opaque with non-toxic white paint. The water surface was above the platform by 1–2 cm. The experiment included training on days 1–4, and the training was performed twice daily. A training trial was completed when the mouse found a platform within 60 s and after the trial, the mouse remained on the platform for 30 s after climbing onto it. On day 5, spatial exploration capability was tested. The platform was removed and the mice were placed in the first quadrant to record the number of times they crossed the original platform position within the 60 s. Mice with cognitive deficits should cross the platform less often during the exploration experiments.

### 4.6. Molecular Analyses of Mouse Hippocampus Tissue

#### 4.6.1. ELISA 

Differences in S1P levels in mouse hippocampal tissues and serum were determined by the S1P Elisa Kit (Echelon Biosciences, Salt Lake City, UT, USA). The experimental operation was performed according to the manufacturer’s instructions. Standard curves were generated to calculate the concentration value of each sample based on the sample protein content (μ mol S1P/mg protein), and the concentrations of S1P in the serum and hippocampal tissues were expressed in molar quantities.

#### 4.6.2. RT-qPCR

Total RNA was extracted from the hippocampus of the mice using RNAiso Plus (Takara Bio, Dalian, China), isopropanol, and 75% ethanol. RNase-free water was finally added to dissolve the RNA. The Nanodrop 2000 spectrophotometer was used to determine the concentration and purity of the total RNA with the OD260/OD280 ratio of >1.9 as the criteria. For reverse transcription, the PrimeScriptTMRT Reagent Kit with gDNA Eraser (Takara Bio, Dalian, China) was used according to the instructions provided by the manufacturer. Real-time RT-qPCR analysis was performed in a LightCycler480 System with the TB Green^®^ Premix Ex TaqTM II (Takara Bio, Dalian, China). The amplification and melting curves of real-time qPCR were confirmed after the reaction. Each sample’s relative expression level was calculated using the 2^−∆∆CT^ method. The primer sequences used in this experiment are listed in Table S1.

#### 4.6.3. Western Blotting 

A total of 30 μg of protein was loaded onto and separated on precast gels, transferred to polyvinylidene fluoride (PVDF) membranes, and probed with antibodies. Horseradish was used to detect the immunoreactive bands and peroxidase-conjugated secondary antibodies were visualized with enhancement. Bands were detected and quantified using Image-Pro Plus software by chemiluminescence. The experiments were repeated at least three times. The antibodies used in this experiment were listed as follows: ApoA-I (Cat. 66206-1-AP, RRID: AB_2881597, 1:2000, Proteintech, Rosemont, IL, USA), SphK1 (Cat. 10670-1-AP, RRID: AB_2195809, 1:1000, Proteintech), SphK2 (Cat. 17096-1-AP, RRID: AB_10598479, 1:3000, Proteintech), phospho-p38 MAPK (Cat. 4511S, RRID: AB_2139682, 1:1000; Cell Signaling, Danvers, MA, USA), p38 MAPK (Cat. 8690S, RRID: AB_10999090, 1:1000, Cell Signaling), phospho-p44/42 MAPK (Cat. 4370S, RRID: AB_2315112, 1:2000, Cell Signaling), p44/42 MAPK (Erk1/2) (Cat. 4695s, RRID: AB_390779, 1:2000, Cell Signaling), CDK5 (D1F7M) (Cat. 14145s, RRID: AB_2773717, 1:1000, Cell Signaling), GAD1 (D1F2M) (Cat. 41318s, RRID: AB_2799198, 1:1000, Cell Signaling), Bax (D3R2M) (Cat. 14796s, RRID: AB_2716251, 1:1000, Cell Signaling), Bcl-2 (D17C4) (Cat. 3498s, RRID: AB_1903907, 1:1000; Cell Signaling), caspase-3 (Cat. 14220s, RRID: AB_2798429, 1:1000, Cell Signaling), phospho-CaMKII (Thr286) (D21E4) (Cat. 12716s, RRID: AB_2713889, 1:1000, Cell Signaling), CaMKII-α (D10C11) (Cat. 11945s, RRID: AB_2797775, 1:1000, Cell Signaling), CREB (Cat. 9197s, RRID: AB_331277, 1:1000, Cell Signaling), phospho-CREB (Cat. 9198s, RRID: AB_2561044, 1:1000, Cell Signaling), KCNQ2 (Cat. AGP-065, RRID: AB_2827304, 1:200; Alomone), KCNQ3 (Cat. APC-051, RRID: AB_2040103, 1:200; Alomone), KCNQ5 (Cat. APC-155, RRID: AB_2341038, 1:200; Alomone) β-actin (Cat. AC026, RRID: AB_2768234, 1:50000, ABclonal), β-tubulin (Cat. 10094-1 AP, RRID: AB_2210695, 1:5000, Proteintech) and GAPDH (Cat. 10494-1-AP, RRID: AB_2263076, 1:5000, Proteintech).

### 4.7. Data Processing and Statistical Analysis

Statistical analysis of the data was performed with SPSS 26 (IBM SPSS Statistics for Windows, version 26.0, Armonk, NY, USA: IBM Corp). The data were described as mean ± SEM, with a significance threshold of α = 0.05 (NS *p* > 0.05, * *p* < 0.05, ** *p* < 0.01, *** *p* < 0.001; **** *p* < 0.0001). The Shapiro–Wilk test was used to assess the normality of the data distribution. The Student’s *t* test was used for comparisons between the two groups. Analyses using one-way ANOVA were used for comparisons between the three groups. If there was a violation of variance homogeneity, Welch’s correction was applied. If there was a violation of normality criteria in the normality and lognormality tests, non-parametric tests were used, and the Kruskal–Wallis test was used for between-group comparisons.

To obtain unbiased data, only the investigator administering the treatment was unblinded to the treatment information. The investigators that performed the behavioral analysis and biochemical analysis were blinded to the treatment grouping.

## 5. Conclusions

In ASD pathogenesis, lipid metabolism may play an important role [66], but the molecular mechanisms are poorly defined. Our findings first revealed that ApoA-I is a hub protein with regard to the lipid metabolism in ASD, where aberrantly upregulated ApoA-I has a negative impact on the core phenotypes of ASD. The mechanism of ApoA-I action on ASD may involve the activation of downstream SphK, which regulates proteins related to the development and function of neuronal synapses. Our findings regarding the effects and potential mechanisms of ApoA-I in ASD will provide a new target and insights into clinical therapeutic strategies for patients with ASD.

## Data Availability

The data that support the findings of this study are available from the corresponding authors upon reasonable request. All supporting data are present in the manuscript.

## Supplementary Materials

The following supporting information can be downloaded at: https://www.mdpi.com/article/10.3390/ijms232315290/s1.
