# Correction: Li et al. Proteomic-Based Approach Reveals the Involvement of Apolipoprotein A-I in Related Phenotypes of Autism Spectrum Disorder in the BTBR Mouse Model. *Int. J. Mol. Sci.* 2022, *23*, 15290

**DOI:** 10.3390/ijms27073313

**Published:** 2026-04-07

**Authors:** Qi Li, Yaxin Shi, Xiang Li, Yuan Yang, Xirui Zhang, Lisha Xu, Zhe Ma, Jia Wang, Lili Fan, Lijie Wu

**Affiliations:** Department of Children’s and Adolescent Health, Public Health College, Harbin Medical University, Harbin 150081, China; 18603666277@163.com (Q.L.); shiyaxin0@163.com (Y.S.); xiangs_li@163.com (X.L.); yang_yuan0829@126.com (Y.Y.); zxrdyx2015@163.com (X.Z.); xulisha2022@163.com (L.X.); mmazhee@foxmail.com (Z.M.); wangjiahyd@163.com (J.W.)

## Error in Figure

In the original publication [1], there was a mistake in Figure 8 as published. Specifically, the representative blots of Caspase-3 and Bcl-2 in Figure 8a were inadvertently misassembled, and the Bcl-2 quantification in Figure 8b has been updated accordingly. The corrected Figure 8 and its updated legend appear below.

## Text Correction

Following the correction of the Bcl-2 statistical values in Figure 8, the Results section has been updated accordingly. A correction has been made to Section 2.8, Paragraph 1:

To investigate whether high ApoA-I levels were associated with apoptosis, we measured the expression levels of apoptosis-associated proteins, including Caspase-3, Bax, Bcl-2, and cyclin-dependent kinase 5 (CDK5). Compared to the control group, Figure 8a,b showed that the expressions of CDK5 (one-way ANOVA, *p* = 0.0495), pro-apoptotic Caspase-3 (one-way ANOVA, *p* = 0.0077), and Bax (one-way ANOVA, *p* = 0.0164) increased, whereas those of the anti-apoptotic protein Bcl-2 (one-way ANOVA, *p* = 0.0055) decreased in the BTBR group. Compared with the BTBR group, in the BTBR + SKI II group (Figure 8a,b), CDK5 (one-way ANOVA, *p* = 0.0417), Caspase-3 (one-way ANOVA, *p* = 0.0471), and Bax (one-way ANOVA, *p* = 0.0092) were downregulated, whereas Bcl-2 (one-way ANOVA, *p* = 0.0215) was upregulated. All aberrantly regulated apoptosis-related proteins in the hippocampus of BTBR mice improved following SKI II intervention.

The authors state that the scientific conclusions are unaffected. This correction was approved by the Academic Editor. The original publication has also been updated.

## Figures and Tables

**Figure 8:**
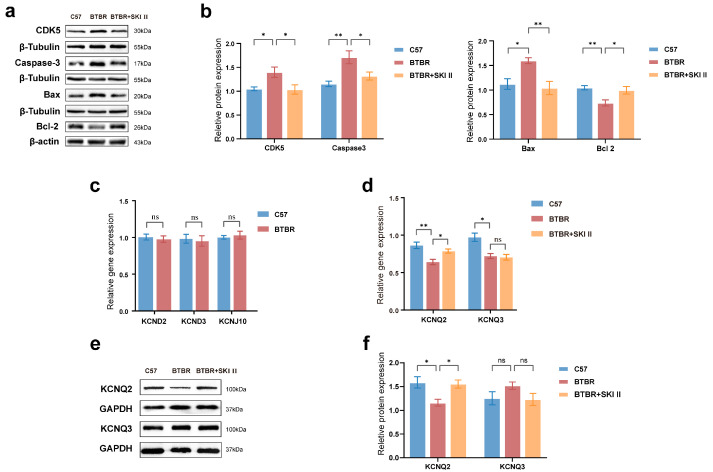
ApoA-I-related pathway regulates the expressions of proteins related to the apoptosis process and the KCNQ2 channel in the hippocampus of the mice. (**a**,**b**) Representative Western blotting bands and quantification of the apoptosis proteins, CDK5/β-tubulin, Caspase-3/β-tubulin, Bax/β-tubulin, and Bcl-2/β-actin ratios. (**c**) Quantification of mRNA expression of KCND2, KCND3, and KCNJ10 channels. (**d**) Quantification of KCNQ2, and KCNQ3 mRNA expression. (**e**,**f**) Representative Western blotting bands and quantification of the M-channel proteins, KCNQ2/GAPDH, and KCNQ3/GAPDH ratios. N = 8–10 per group. All data are shown in bar diagrams, which reflect the arithmetic mean ± standard error of the mean. * *p* < 0.05, ** *p* < 0.01.
