# Supplementary material for: Proteomic-Based Approach Reveals the Involvement of Apolipoprotein A-I in Related Phenotypes of Autism Spectrum Disorder in the BTBR Mouse Model
Source: Int J Mol Sci. 2022 Dec 4;23(23):15290. doi: 10.3390/ijms232315290 (PMC9737945; doi:10.3390/ijms232315290)
Supplement: Supplementary file 1 [file ijms-23-15290-s001.zip › ijms-2002863-supplementary.pdf]

Table S1. Primers used in the present study.

Target	Forward	Reverse
ApoA- Ⅰ	*GCTCAAGAGCAACCCTACCTT*	*GCTTTCTCGCCAAGTGTCTTC*
SphK1	*ACTGATACTCACCGAACGGAA*	*CCATCACCGGACATGACTGC*
SphK2	*ACAGCGACTACGCCCAAAG*	*GTGGGTAGGTGTAGATGCAGA*
Gapdh	*AGGTCGGTGTGAACGGATTTG*	*GGGGTCGTTGATGGCAACA*
KCNQ2	*CGTGACTATCGTGGTATTCGG*	*ACAGCAATGGAGGCAATCAGC*
KCNQ3	*GAGCCGACAAAGACGGGAC*	*TTGGCGTTGTTCCTCTTGACT*
KCNJ10	*GTCGGTCGCTAAGGTCTATTACA*	*GGCCGTCTTTCGTGAGGAC*
KCND2	*GGGTGGATGCCTGTTGCTT*	*GTCTTGCCATGTCTGGAAACG*
KCND3	*GCTCCAGCGGACAAGAACAA*	*CTACCCAGCAAGGTGTCGG*
